# Effect of ertugliflozin on blood pressure in patients with type 2 diabetes mellitus: a post hoc pooled analysis of randomized controlled trials

**DOI:** 10.1186/s12933-019-0856-7

**Published:** 2019-05-07

**Authors:** Jie Liu, Annpey Pong, Silvina Gallo, Amanda Darekar, Steven G. Terra

**Affiliations:** 1 Merck & Co., Inc., Kenilworth, NJ USA; 2 Pfizer Pharma GmbH, Berlin, Germany; 3 Pfizer R&D UK Ltd, Walton Oaks, Tadworth, UK; 4 Pfizer Inc., Andover, MA USA

**Keywords:** Ertugliflozin, Sodium–glucose cotransporter 2 inhibitor, Blood pressure, Systolic blood pressure, Diastolic blood pressure, Pulse rate, Hypertension, Type 2 diabetes mellitus

## Abstract

**Background:**

The efficacy of ertugliflozin, a sodium–glucose cotransporter 2 inhibitor, for glycemic and blood pressure (BP) control has been demonstrated in phase 3 studies. To further evaluate the effects of ertugliflozin on BP and other hemodynamic parameters, an analysis was conducted on the pooled patient populations from these studies.

**Methods:**

This was a post hoc analysis of data from three phase 3 studies (NCT01958671, NCT02033889, and NCT02036515) of adults with type 2 diabetes mellitus who received placebo, ertugliflozin 5 mg, or ertugliflozin 15 mg. Outcomes at 26 weeks were analyzed for the pooled population and according to relevant baseline factors, including BP.

**Results:**

Of the 1544 patients included (placebo, n = 515; ertugliflozin 5 mg, n = 519; ertugliflozin 15 mg, n = 510), most (67.4–69.0%) had hypertension at baseline. Mean baseline BP was similar across treatment groups (placebo, 129.7/78.0 mmHg; ertugliflozin 5 mg, 131.0/78.4 mmHg; ertugliflozin 15 mg, 130.5/78.4 mmHg). At Week 26, placebo-adjusted least squares (LS) mean changes (95% confidence intervals [CI]) from baseline in systolic BP (SBP) were − 3.7 mmHg (− 5.1, − 2.3) for both ertugliflozin doses. Reductions were consistent across all baseline subgroups. At Week 26, more patients with a baseline SBP ≥ 130 mmHg had a SBP < 130 mmHg with ertugliflozin (38.7% both doses) than with placebo (24.0%), and more patients with a baseline SBP ≥ 140 mmHg attained a SBP < 140 mmHg with ertugliflozin (59.5% [5 mg] and 66.7% [15 mg]) than with placebo (43.8%). Placebo-adjusted LS mean changes (95% CI) in diastolic BP (DBP) with ertugliflozin 5 mg and 15 mg were − 1.8 mmHg (− 2.7, − 0.9) and − 1.6 mmHg (− 2.5, −  0.7), respectively, and in pulse rate were − 1.3 beats per minute (bpm) (− 2.2, − 0.3) and − 1.5 bpm (− 2.5, − 0.6), respectively. Greater reductions in pulse pressure, mean arterial pressure, and double product were observed with ertugliflozin than with placebo. Incidence of adverse event-related osmotic diuresis was low, but greater with ertugliflozin (2.9% [5 mg], 2.4% [15 mg]) than placebo (1.0%).

**Conclusion:**

Ertugliflozin treatment led to reductions in SBP, DBP, and pulse rate relative to placebo. Reductions in SBP were generally consistent across the subgroups evaluated.

*Trial registration* NCT01958671; NCT02033889; NCT02036515

**Electronic supplementary material:**

The online version of this article (10.1186/s12933-019-0856-7) contains supplementary material, which is available to authorized users.

## Background

Hypertension is a common comorbidity in patients with type 2 diabetes mellitus (T2DM) and a major risk factor for cardiovascular (CV), cerebrovascular, and renal disease [1–3]. Blood pressure (BP) control is of particular clinical significance as CV disease remains the leading cause of mortality in adults with T2DM [4]. For adults with T2DM, the American Diabetes Association advise a target systolic BP (SBP) < 140 mmHg and diastolic BP (DBP) < 90 mmHg [1], and the European Society of Cardiology recommend a target SBP < 140 mmHg and DBP < 80 mmHg [5]. The American Heart Association guidelines propose a lower target of < 130/80 mmHg in adults with T2DM and hypertension [3], and the European Society of Cardiology guidelines advise that if antihypertensive therapy is tolerated, achieving a SBP < 130 mmHg should be considered due to the benefits of stroke prevention. Despite these guideline recommendations, a substantial number of patients with T2DM do not achieve the recommended BP targets [6–9].

In addition to improved glycemic control, clinically relevant reductions in BP in patients with T2DM have been observed with sodium–glucose cotransporter 2 (SGLT2) inhibitor treatment [10–13]. Beneficial effects on BP were also observed in SGLT2 inhibitor CV outcomes studies [14–18].

Reductions in SBP were observed with ertugliflozin, a highly selective SGLT2 inhibitor, in the eValuation of ERTugliflozin effIcacy and Safety (VERTIS) phase 3 clinical trials program in patients with T2DM [19–23]. In order to characterize the BP-lowering effect of ertugliflozin and to examine the influence of BP-related factors, such as age, ethnicity, hypertension status, and use of antihypertensive agents on BP and other related outcomes, we conducted a post hoc analysis of pooled patient data from three placebo-controlled phase 3 clinical studies in the VERTIS program [19, 20, 23]. These studies were considered suitable for such an analysis as they included similar patient populations and had similar designs [19, 20, 23]. In addition to characterizing the BP-lowering effects of ertugliflozin, we evaluated the incidence of adverse events (AEs), including events related to osmotic diuresis and hypovolemia. The proportion of patients with orthostatic changes was also analyzed. Here, we present the results of this analysis.

## Methods

### Data sources

Data were pooled from VERTIS MONO (protocol MK-8835-003; clinicaltrials.gov identifier NCT01958671) [23], VERTIS MET (protocol MK-8835-007; clinicaltrials.gov identifier NCT02033889) [19], and VERTIS SITA2 (protocol MK-8835-006; clinicaltrials.gov identifier NCT02036515) [20]. All studies contributing data to this analysis were conducted in accordance with principles of Good Clinical Practice, and were approved by the appropriate institutional review boards and regulatory agencies. Informed consent was obtained from individuals in each study. The analyses in this article are based on previously conducted studies and do not involve any new studies of human patients or animal subjects performed by any of the authors. Methods and results of the three individual studies have been reported previously [19, 20, 23], and the studies are briefly described below.

### Patients and data sources

Adults with T2DM according to the American Diabetes Association criteria [24] with baseline glycated hemoglobin (HbA1c) levels of 7.0% to 10.5% (inclusive) were enrolled. Key exclusion criteria included: type 1 diabetes mellitus; history of ketoacidosis; estimated glomerular filtration rate (eGFR) < 55 mL/min/1.73 m^2^ (< 60 mL/min/1.73 m^2^ in VERTIS SITA2); history of a CV event within 3 months of screening; and mean value for triplicate of sitting SBP > 160 mmHg and/or DBP > 90 mmHg at any time during screening (patients receiving BP medication must have had a stable regimen for ≥ 4 weeks prior to randomization) [19, 20, 23].

All studies comprised a 26-week, double-blind, placebo-controlled treatment period (phase A), followed by a phase B treatment period of 26 weeks (78 weeks for VERTIS MET [19]). The primary efficacy time point of the three individual studies was Week 26; data up to this time point are included in the present analysis.

Patients were randomized 1:1:1 to placebo, ertugliflozin 5 mg, or ertugliflozin 15 mg once daily. Patients in VERTIS MONO received no concomitant background antihyperglycemic therapy [23]; patients in VERTIS MET received concomitant metformin monotherapy (≥ 1500 mg/day) [19]; and patients in VERTIS SITA2 received concomitant metformin (≥ 1500 mg/day) and sitagliptin (100 mg/day) [20].

### Endpoints and assessments

The efficacy endpoints reported for this post hoc analysis were the change from baseline in SBP, DBP, pulse rate, pulse pressure (SBP–DBP), mean arterial pressure (calculated as 2/3 DBP + 1/2 SBP) and double product (SBP × pulse rate) at Week 26. The percentage of patients with SBP  < 130 mmHg (among patients with baseline SBP ≥ 130 mmHg) and SBP < 140 mmHg (among patients with baseline SBP ≥ 140 mmHg) at Week 26 were also reported. Use of antihypertensive therapy at baseline and at Week 26 was also reported.

In each study, sitting BP was measured in triplicate with an automated oscillometric BP measuring device, with measurements after at least 5 min of rest. Patients were advised to avoid nicotine-containing products and/or ingesting caffeine for at least 30 min preceding the measurements.

A summary of the incidence of AEs is reported, as well as the incidences of orthostatic change in SBP and DBP, and the incidence of AEs related to osmotic diuresis and hypovolemia. Orthostatic change in SBP and DBP was defined as a reduction (after 1 and/or 3 min of standing from the supine position) of ≥ 20 mmHg or ≥ 10 mmHg, respectively.

### Statistical methods

The primary efficacy analyses were conducted on the population of all randomized, treated patients who had at least one measurement of the analysis endpoint at or after baseline. The safety analysis was conducted on all randomized, treated patients. All analyses (efficacy and safety) included data obtained after the initiation of glycemic rescue therapy. As this was a post hoc exploratory analysis, no pre-specified hypotheses were tested, and p values are not presented (except for assessing the significance of correlation coefficients).

The primary analysis of changes from baseline in sitting SBP, DBP, pulse rate, pulse pressure, mean arterial pressure, and double product used a longitudinal data analysis model with fixed effects to adjust for treatment, time, study, baseline eGFR, and the interaction of time by treatment [25]. Time was treated as a categorical variable. No imputation of missing data was performed. The differences in least squares (LS) means with 95% confidence interval (CI) for comparisons of ertugliflozin 5 mg or 15 mg versus placebo were calculated.

The percentage of patients at Week 26 with SBP < 130 mmHg (among patients with SBP ≥ 130 mmHg at baseline) and < 140 mmHg (among patients with a baseline SBP ≥ 140 mmHg) compared with placebo were analyzed using the Miettinen and Nurminen method [26]; patients with missing data at Week 26 were considered non-responders.

The following subgroups were analyzed for change from baseline in SBP using a repeated measures analysis of covariance model: age (< 65 or ≥ 65 years), sex, race (White, Black, Asian, or other), baseline body mass index (BMI; above or below median [30.8 kg/m^2^]), baseline eGFR (< 90 or ≥ 90 mL/min/1.73 m^2^), baseline SBP (≤ 130, or > 130–140, or > 140 mmHg), baseline antihypertensive therapy use (diuretic and/or renin–angiotensin–aldosterone system [RAAS] blocker use), and baseline HbA1c level (< 8.0%, 8.0% to < 9.0%, or ≥ 9.0%). The analysis of covariance model was adjusted for treatment, time, study, baseline eGFR, baseline value of the response variable, and the interaction of time by treatment. The population for subgroup analyses contained all randomized, treated patients who had a baseline SBP measurement and at least one SBP measurement after baseline.

The number and percentage of patients with antihypertensive therapy use at baseline and at Week 26 are presented by treatment group.

Scatter plots of individual participant data presented change from baseline in SBP at Week 26 versus change in HbA1c at Week 26 and change from baseline in body weight at Week 26. Pearson correlation coefficients were used to test the linear relationship between the variables.

## Results

### Patient population

A total of 1544 randomized patients were included in the analyses (461 from VERTIS MONO, 621 from VERTIS MET, and 462 from VERTIS SITA2). Of these, 515 patients received placebo, 519 ertugliflozin 5 mg, and 510 ertugliflozin 15 mg. Patient demographic and baseline clinical characteristics were balanced across treatment groups (Table 1). At baseline, 67.8%, 69.0%, and 67.4% of patients had a history of hypertension in the placebo, ertugliflozin 5 mg, and ertugliflozin 15 mg groups, respectively. The corresponding mean SBP/DBP values at baseline were 129.7/78.0 mmHg, 131.0/78.4 mmHg, and 130.5/78.4 mmHg, respectively. At baseline, 60.8%, 62.8%, and 60.4% of patients were using antihypertensive therapies in the placebo, ertugliflozin 5 mg, and ertugliflozin 15 mg groups, respectively. The majority of these were RAAS blockers, which were used in 54.8%, 56.5%, and 54.5% of patients at baseline in the placebo, ertugliflozin 5 mg and ertugliflozin 15 mg groups, respectively.

**Table 1:** Baseline demographics and clinical characteristics

	Placebo (n = 515)	Ertugliflozin 5 mg (n = 519)	Ertugliflozin 15 mg (n = 510)
Age, years	56.9 (9.6)	57.4 (9.6)	57.5 (9.7)
Male, n (%)	280 (54.4)	267 (51.4)	265 (52.0)
BMI, kg/m^2^	31.3 (6.0)	31.6 (6.0)	31.5 (5.4)
Race, n (%)			
White	378 (73.4)	382 (73.6)	374 (73.3)
Asian	79 (15.3)	77 (14.8)	77 (15.1)
Black or African American	31 (6.0)	34 (6.6)	37 (7.3)
Other	27 (5.2)	26 (5.0)	22 (4.3)
Duration of T2DM, years	7.4 (5.9)	7.6 (6.1)	7.6 (5.7)
HbA1c, %^a^	8.1 (0.9)	8.1 (0.9)	8.2 (1.0)
eGFR, mL/min/1.73 m^2^	89.5 (19.1)	88.2 (17.7)	89.0 (18.5)
Medical history of hypertension, n (%)	349 (67.8)	358 (69.0)	344 (67.4)
Sitting SBP, mmHg^b^			
Mean (SD)	129.7 (14.5)	131.0 (13.3)	130.5 (13.0)
≤ 130, n (%)	268 (53.2)	244 (47.7)	249 (49.6)
> 130, n (%)	236 (46.8)	268 (52.3)	253 (50.4)
≤ 140, n (%)	388 (77.0)	392 (76.6)	383 (76.3)
> 140, n (%)	116 (23.0)	120 (23.4)	119 (23.7)
Sitting DBP, mmHg^b^	78.0 (7.5)	78.4 (7.9)	78.4 (7.5)
Pulse rate, bpm	72.6 (9.2)	72.8 (10.0)	72.6 (9.3)
Microvascular disease^c^, n (%)	85 (16.5)	90 (17.3)	86 (16.9)
Antihypertensive therapy used, n (%)^d^			
Any	313 (60.8)	326 (62.8)	308 (60.4)
Diuretics	31 (6.0)	41 (7.9)	38 (7.5)
RAAS blockers	282 (54.8)	293 (56.5)	278 (54.5)
β blockers	127 (24.7)	113 (21.8)	105 (20.6)
Calcium channel blockers	12 (2.3)	10 (1.9)	13 (2.6)

Data presented as mean (SD), unless otherwise specified

*BMI* body mass index, *bpm* beats per minute, *DBP* diastolic blood pressure, *eGFR* estimated glomerular filtration rate, *HbA1c* glycated hemoglobin, *RAAS* renin–angiotensin–aldosterone system, *SBP* systolic blood pressure, *SD* standard deviation, *T2DM* type 2 diabetes mellitus

^a^Number of patients with data: 512 (placebo), 515 (ertugliflozin 5 mg), 504 (ertugliflozin 15 mg)

^b^Number of patients with data: 504 (placebo), 512 (ertugliflozin 5 mg), 502 (ertugliflozin 15 mg)

^c^Included preferred terms defined by a sponsor-generated custom Medical Dictionary for Regulatory Activities (MeDRA) query reported as medical history related to diabetic microvascular complications (Additional file 1)

^d^Some patients took more than one hypertension therapy at baseline

### BP and pulse rate

**Fig. 1:**
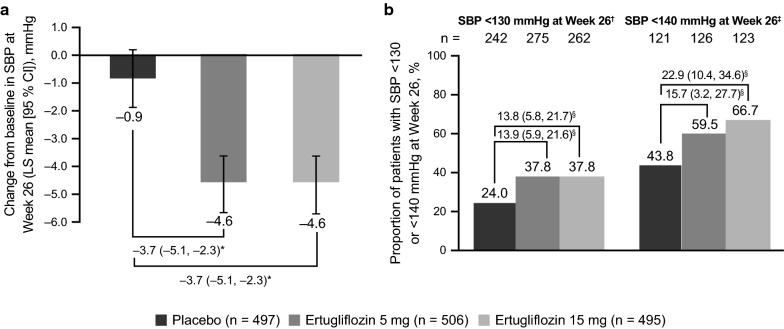
Change from baseline in systolic blood pressure (SBP). Change from baseline in SBP at Week 26 (**a**) and proportion of patients with SBP < 130 mmHg and < 140 mmHg at Week 26 (**b**). *CI* confidence interval; *LS* least squares. *Placebo-adjusted difference in LS mean (95% CI). ^†^Of patients with baseline SBP of ≥ 130 mmHg. ^‡^Of patients with baseline SBP of ≥ 140 mmHg. ^§^Difference in response rate (95% CI)

Treatment with ertugliflozin 5 mg and 15 mg resulted in a greater reduction from baseline in SBP at Week 26 compared with placebo (placebo-adjusted LS mean changes [95% CI] from baseline in SBP were − 3.7 mmHg [− 5.1, − 2.3] for both ertugliflozin doses; Fig. 1a).

The proportion of patients with SBP ≥ 130 mmHg at baseline who subsequently achieved SBP < 130 mmHg at Week 26 was higher in the ertugliflozin 5 mg and 15 mg groups compared with the placebo group (37.8% with both ertugliflozin doses versus 24.0% with placebo; Fig. 1b). At Week 26, 59.5% and 66.7% of patients with baseline SBP ≥ 140 mmHg achieved a SBP < 140 mmHg in the ertugliflozin 5 mg and 15 mg groups, respectively, versus 43.8% of patients in the placebo group (Fig. 1b).

**Fig. 2:**
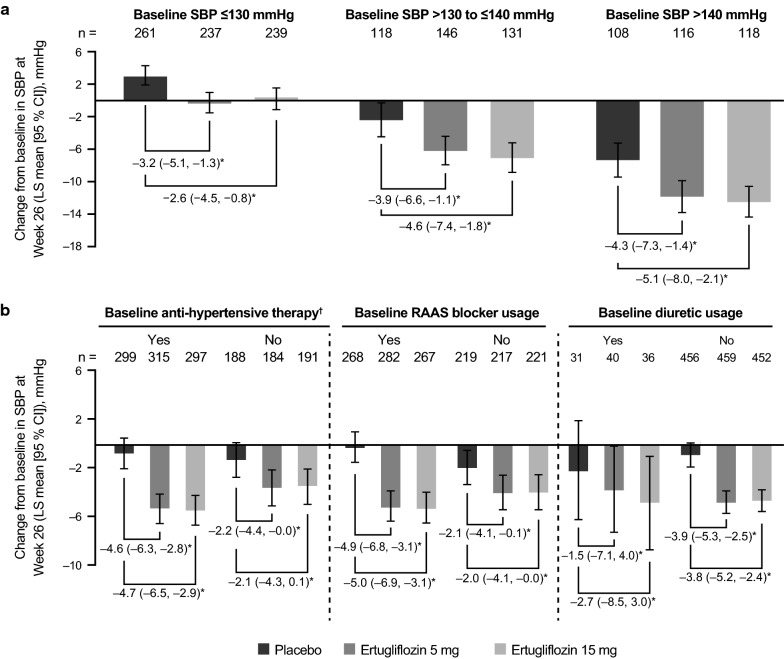
Change from baseline in systolic blood pressure (SBP) by baseline SBP and antihypertensive therapy use. Change from baseline in SBP at Week 26 by baseline SBP level (**a**) and baseline antihypertensive therapy, diuretics, and renin–angiotensin–aldosterone system (RAAS) blocker use (**b**). *CI* confidence interval; *LS* least squares. *Placebo-adjusted difference in LS mean (95% CI). ^†^Mean baseline SBP across groups was 126–127 or 132–133 mmHg in patients with or without baseline antihypertensive therapy, respectively

**Fig. 3:**
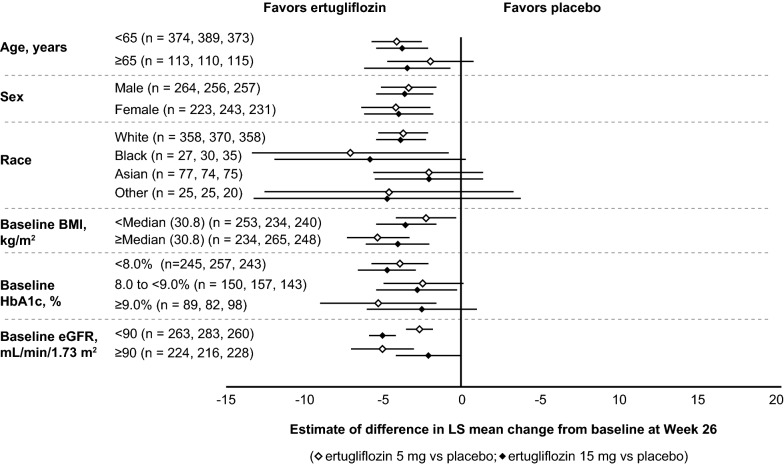
Estimate of difference from baseline in systolic blood pressure (SBP) at Week 26 by subgroup. Data are presented as n_1_, n_2_, and n_3_ where n_1_ = number of patients in the placebo group, n_2_ = number of patients in the ertugliflozin 5 mg group, and n_3_ = number of patients in the ertugliflozin 15 mg group. *BMI* body mass index, *eGFR* estimated glomerular filtration rate, *HbA1c* glycated hemoglobin

Patients with a high baseline SBP (> 130 to ≤ 140 mmHg and > 140 mmHg) exhibited larger LS mean reductions from baseline in SBP compared with patients with low baseline SBP values (≤ 130 mmHg) across treatment groups. Furthermore, larger LS mean reductions from baseline in SBP were demonstrated in patients receiving ertugliflozin compared with placebo in all baseline SBP subgroups (Fig. 2a). In general, LS mean reductions in SBP from baseline were greater in the ertugliflozin groups than in the placebo group across all subgroups by baseline SBP (Fig. 2a). In patients with baseline antihypertensive therapy or RAAS blocker use, both ertugliflozin 5 mg and 15 mg led to marked reductions in SBP compared with placebo (Fig. 2b). Reductions in SBP were not meaningfully different in the ertugliflozin groups compared with placebo in patients taking a diuretic at baseline. However, the low number of patients in each treatment group within this subgroup resulted in a reduced precision of the estimates (Fig. 2b). Ertugliflozin resulted in placebo-adjusted SBP reductions from baseline in the patient subgroups, including patients with an eGFR < 90 mL/min/1.73 m^2^ (Fig. 3).

**Fig. 4:**
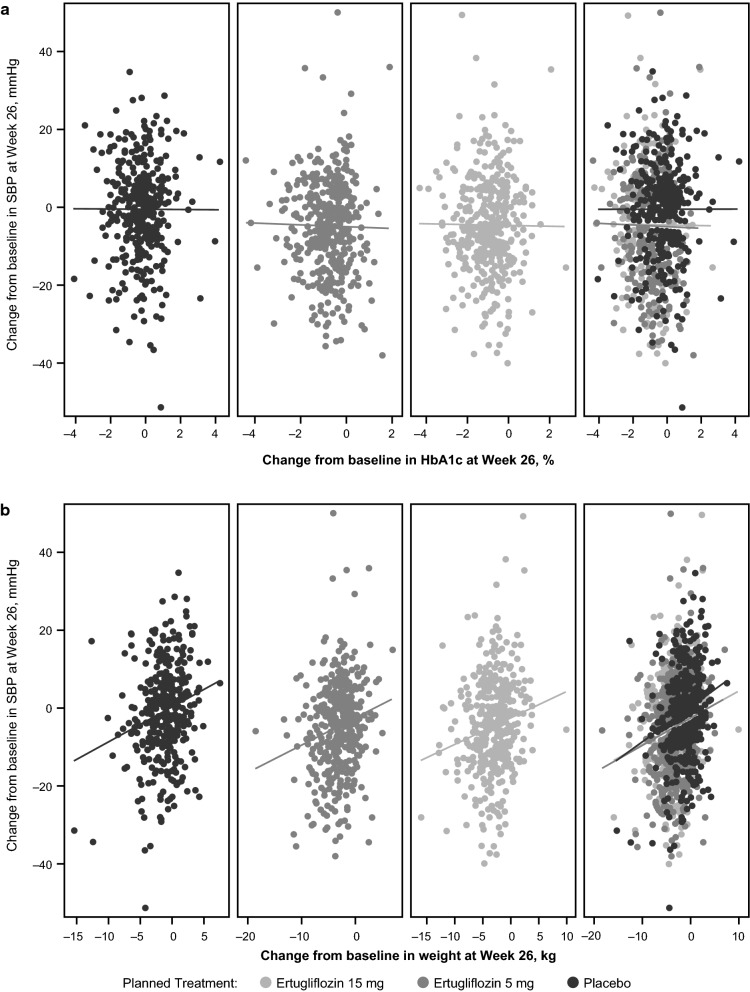
Correlation between systolic blood pressure (SBP) and glycated hemoglobin (HbA1c) and body weight. Change from baseline in SBP at Week 26 versus change from baseline in HbA1c at Week 26 (**a**) and change from baseline in body weight at Week 26 (**b**). *HbA1c* glycated hemoglobin

No correlations between changes from baseline in SBP and HbA1c at Week 26 were observed in the placebo or ertugliflozin groups (Pearson correlation coefficient p values > 0.05; Fig. 4a). Across all treatment groups, there was a significant correlation between the change from baseline in SBP and body weight at Week 26 (Pearson correlation coefficient p values < 0.05; Fig. 4b).

**Fig. 5:**
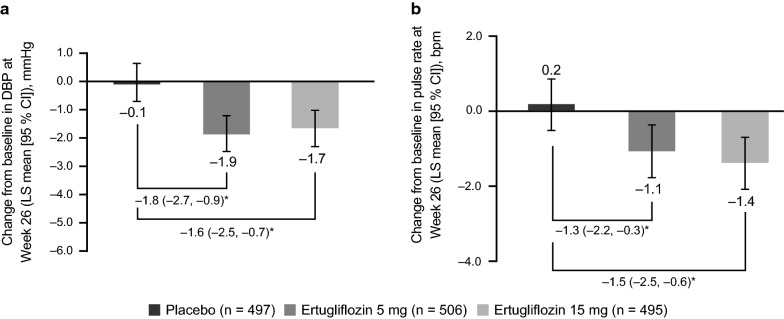
Change from baseline in sitting diastolic blood pressure (DBP) and pulse rate at Week 26. Change from baseline in sitting DBP at Week 26 (**a**) and change from baseline in sitting pulse rate at Week 26 (**b**). *CI* confidence interval, *LS* least squares. *Placebo-adjusted difference in LS mean (95% CI)

Both ertugliflozin 5 mg and 15 mg resulted in a greater reduction from baseline in DBP and pulse rate compared with placebo at Week 26 (Fig. 5a, b).

### Other BP parameters and antihypertensive therapy use

Compared with placebo, greater reductions from baseline in pulse pressure, mean arterial pressure, and double product were observed in both the ertugliflozin 5 mg and 15 mg groups (Table 2).

**Table 2:** Change from baseline in other blood pressure parameters at Week 26

	Placebo (n = 504)	Ertugliflozin 5 mg (n = 519)	Ertugliflozin 15 mg (n = 510)
Pulse pressure, mmHg^a^			
Baseline mean (SD)^b^	51.8 (12.1)	52.5 (11.3)	52.1 (11.2)
LS mean change from baseline (95% CI)	− 0.8 (− 1.7, 0.0)	− 2.7 (− 3.5, − 1.9)	− 2.9 (− 3.8, − 2.1)
Placebo-adjusted LS mean change from baseline (95% CI)	–	− 1.9 (− 3.0, − 0.8)	− 2.1 (− 3.3, − 1.0)
Mean arterial pressure, mmHg^a^			
Baseline mean (SD)^b^	95.2 (8.7)	96.0 (8.5)	95.8 (8.1)
LS mean change from baseline (95% CI)	− 0.3 (− 1.0, 0.4)	− 2.8 (− 3.4, − 2.1)	− 2.65 (− 3.3, − 2.0)
Placebo-adjusted LS mean change from baseline (95% CI)	–	− 2.4 (− 3.4, − 1.5)	− 2.3 (− 3.2, − 1.4)
Double product, mmHg × bpm^a^			
Baseline mean (SD)^c^	9409.2 (1536.9)	9544.7 (1668.6)	9480.5 (1571.1)
LS mean change from baseline (95% CI)	− 44.0 (− 161.7, 73.7)	− 479.9 (− 594.5, − 365.3)	− 514.1 (− 630.8, − 397.4)
Placebo-adjusted LS mean change from baseline (95% CI)	–	− 435.9 (− 593.6, − 278.2)	− 470.0 (− 629.3, − 310.8)

*bpm* beats per minute, *CI* confidence interval, *LS* least squares

^a^Number of patients with data at Week 26: 497 (placebo), 506 (ertugliflozin 5 mg), 495 (ertugliflozin 15 mg)

^b^Number of patients with data: 504 (placebo), 512 (ertugliflozin 5 mg), 502 (ertugliflozin 15 mg)

^c^Number of patients with data: 504 (placebo), 512 (ertugliflozin 5 mg), 501 (ertugliflozin 15 mg)

There was no meaningful change from baseline in the use of antihypertensive therapies across groups at Week 26 (Table 3).

**Table 3:** Antihypertensive therapy use at baseline and Week 26

Time point	Placebo	Ertugliflozin 5 mg	Ertugliflozin 15 mg
Patients with one or more antihypertensive therapies			
Baseline^a^	337 (65.4)	353 (68.0)	336 (65.9)
Week 26^b^	316 (66.8)	344 (69.5)	318 (67.5)
RAAS blockers			
Baseline^a^	292 (56.7)	306 (59.0)	289 (56.7)
Week 26^b^	278 (58.8)	298 (60.2)	271 (57.5)
β blockers			
Baseline^a^	120 (23.3)	104 (20.0)	98 (19.2)
Week 26^b^	113 (23.9)	101 (20.4)	96 (20.4)
Calcium channel blockers			
Baseline^a^	94 (18.3)	91 (17.5)	116 (22.7)
Week 26^b^	88 (18.6)	93 (18.8)	108 (22.9)
Diuretics			
Baseline^a^	106 (20.6)	112 (21.6)	104 (20.4)
Week 26^b^	99 (20.9)	106 (21.4)	98 (20.8)
Other antihypertensive therapy			
Baseline^a^	22 (4.3)	18 (3.5)	18 (3.5)
Week 26^b^	22 (4.7)	18 (3.6)	18 (3.8)

Every patient is counted a single time for each applicable specific medication. A patient with multiple medications within a medication category is counted a single time for that category. Data presented as number of patients (%)

*RAAS* renin–angiotensin–aldosterone system

^a^Number of patients with data: 515 (placebo), 519 (ertugliflozin 5 mg), and 510 (ertugliflozin 15 mg)

^b^Number of patients with data: 473 (placebo), 495 (ertugliflozin 5 mg), and 471 (ertugliflozin 15 mg)

### Safety

The incidence of AEs was similar across treatment groups (Table 4). The incidence of serious AEs and AEs that led to discontinuation of study medication was low and not meaningfully different between groups. The incidence of hypovolemia AEs, including hypotension, was low across the treatment groups. The incidence of AEs of osmotic diuresis was low across the treatment groups, but higher in the ertugliflozin groups relative to the placebo group. The proportions of patients who met the pre-specified definition for orthostatic changes in SBP and DBP were similar between treatment groups at baseline, Week 6, and Week 26 (Table 4).

**Table 4:** Summary of overall safety and orthostatic blood pressure changes

	Placebo (n = 515)	Ertugliflozin 5 mg (n = 519)	Ertugliflozin 15 mg (n = 510)
Any AE	263 (51.1)	236 (45.5)	257 (50.4)
Serious AE	15 (2.9)	17 (3.3)	12 (2.4)
Discontinuation due to AE^a^	9 (1.7)	12 (2.3)	7 (1.4)
Hypovolemia	9 (1.7)	4 (0.8)	5 (1.0)
Osmotic diuresis	5 (1.0)	15 (2.9)	12 (2.4)
Orthostatic change in SBP			
Baseline, n/m (%)	14/502 (2.8)	21/516 (4.1)	16/494 (3.2)
Week 6, n/m (%)	16/477 (3.4)	16/493 (3.2)	17/476 (3.6)
Week 26, n/m (%)	18/446 (4.0)	16/475 (3.4)	17/458 (3.7)
Orthostatic change in DBP			
Baseline, n/m (%)	72/502 (14.3)	80/516 (15.5)	81/494 (16.4)
Week 6, n/m (%)	74/477 (15.5)	81/493 (16.4)	68/476 (14.3)
Week 26, n/m (%)	65/446 (14.6)	82/475 (17.3)	75/458 (16.4)

Data presented as number of patients (%). n is the number of patients with test results at that visit that met the predetermined criterion; m is the number of patients with at least one test result at that visit

*AE* adverse event, *DBP* diastolic blood pressure, *SBP* systolic blood pressure

^a^Study medication withdrawn

## Discussion

In this post hoc analysis of data from three phase 3 randomized, placebo-controlled studies, ertugliflozin 5 mg and 15 mg resulted in greater reductions in SBP after 26 weeks of treatment compared with placebo. These reductions were observed across the subgroups analyzed. Greater mean reductions in DBP and pulse rate were also observed with ertugliflozin compared with placebo. The effects on BP in this pooled analysis are generally consistent with results from studies with other SGLT2 inhibitors [11, 12, 27–31]. Greater reductions in BP with ertugliflozin relative to placebo over a longer treatment period of 104 weeks have also been reported [32]. In addition, a meta-analysis of SGLT2 inhibitor effects on BP reported significant placebo-adjusted changes (weighted mean difference) of − 2.5 mmHg and − 1.5 mmHg in SBP and DBP, respectively [33]. Reductions in SBP with ertugliflozin were also observed in active comparator studies [21, 34]. For example, compared with glimepiride, ertugliflozin lowered SBP by − 3.2 mmHg (ertugliflozin 5 mg) and − 4.8 mmHg (ertugliflozin 15 mg) at Week 52 [34]. Similarly, the VERTIS FACTORIAL study reported favorable changes in SBP at Week 26 in the ertugliflozin treatment groups (− 3.9 mmHg [ertugliflozin 5 mg] and − 3.7 mmHg [ertugliflozin 15 mg]) compared with sitagliptin 100 mg (− 0.7 mmHg).

The change from baseline in placebo-adjusted, clinic-measured SBP was also similar to the lowering of SBP observed with ertugliflozin in a study that utilized 24-hour ambulatory BP monitoring (− 3.0 to − 4.0 mmHg) [35], and in a systematic review and meta-analysis of SGLT2 inhibitor effects on 24-h ambulatory SBP (− 3.8 mmHg) [36].

In the current study, greater reductions in SBP in all treatment groups (ertugliflozin and placebo groups) were seen in patients with higher versus lower baseline SBP. Furthermore, patients receiving ertugliflozin were more likely to achieve guideline-recommended SBP goals at Week 26 than patients receiving placebo. This study and many others have shown the benefits of ertugliflozin and other SGLT2 inhibitors not only on glycemic control but also on BP reduction [19–22, 29, 30, 34]. Lowering elevated BP to the guideline-recommended targets in adults with T2DM is associated with reductions in macrovascular and microvascular complications, and death [5].

Ertugliflozin reduced SBP relative to placebo in patients receiving baseline standard of care antihypertensive therapy, including in patients using RAAS inhibitors. RAAS activity, which is a determining factor of BP, may be reduced in response to SGLT2 inhibition, mediating an increase in sodium delivery to the macula densa. However, the mechanism by which ertugliflozin reduces BP is likely to be multifactorial [36]. Changes from baseline in SBP did not correlate with changes from baseline in HbA1c in this analysis. This suggests that glycosuria (and therefore osmotic diuresis) is not the sole mechanism accounting for the BP reductions reported here, a finding also suggested in studies with other SGLT2 inhibitors [11–13, 37]. Alternative pathways may account for the BP-lowering effects reported with ertugliflozin and other SGLT2 inhibitors, such as reductions in arterial stiffness and weight loss [10, 38]. In keeping with this, changes from baseline in SBP did correlate with changes from baseline in body weight in both placebo and ertugliflozin groups in this analysis. Other studies have also shown that reductions in body weight were associated with reductions in arterial stiffness and BP [38]. Treatment with SGLT2 inhibitors can alter body composition by reducing total body fat with minimal changes to lean muscle; reduction in body fat has been suggested as an additional mechanism by which SGLT2 inhibitors lower BP [36]. However, a rodent model of an SGLT2 inhibitor (empagliflozin) reported that reductions in arterial stiffness were not associated with improvements in BP [39]. Furthermore, studies of ertugliflozin have demonstrated that the reductions in SBP from baseline are observed early, by Week 12 to Week 18, and are then maintained, whereas placebo-adjusted reductions in weight from baseline plateau at the later time point of 26 weeks [20, 34], suggesting that factors beyond weight loss contribute to BP lowering.

The reductions in pulse pressure, mean arterial pressure, and double product reported here are also consistent with studies in other SGLT2 inhibitors [11, 40]. Reductions in pulse and mean arterial pressure may lead to beneficial effects on arterial stiffness and cardiac workload [41]. Despite the natriuretic and volume depletion effects of ertugliflozin, pulse rate was reduced compared with placebo. This suggests autonomic nervous system regulation with ertugliflozin, a hypothesis speculated in response to similar findings with other SGLT2 inhibitors [10].

Improvements in composite CV outcomes have now been demonstrated with three SGLT2 inhibitors [14, 15, 18]. Although the mechanisms through which SGLT2 inhibitors confer the observed CV benefits are not clear, the BP-lowering effects of SGLT2 inhibitors may be a relevant factor. Control of BP has been shown to reduce the excess risk of CV outcomes (death, stroke, myocardial infarction, and hospitalization for heart failure) in patients with T2DM [42]. The long-term effects of ertugliflozin on CV outcomes are being assessed in the VERTIS Cardiovascular Outcomes study (VERTIS CV; NCT01986881), a randomized, double-blind, placebo-controlled study in patients with T2DM and established atherosclerotic CV disease [43].

In this analysis, both doses of ertugliflozin were generally well tolerated, with an overall safety profile similar to placebo. It has been suggested that treatment with SGLT2 inhibitors may contribute to diuresis-induced hypovolemia [11, 12, 44, 45], and it could be speculated that this might add to risks from the use of antihypertensive therapies in this patient population. While the use of some BP-lowering agents in patients with T2DM is associated with orthostatic hypotension [46], BP reductions in the ertugliflozin groups were not accompanied by an increase from baseline in the incidence of orthostatic changes in SBP and DBP. The incidence of AEs related to osmotic diuresis was higher in the ertugliflozin groups compared with placebo as expected in this class. Considering that baseline diuretic use was low and balanced among treatment groups (6.8%, 7.9%, and 7.5% for placebo, ertugliflozin 5 mg, and ertugliflozin 15 mg, respectively), the higher incidence of AEs related to osmotic diuresis observed in the ertugliflozin groups is likely due to ertugliflozin, as expected by its mechanism of action. The incidence of hypovolemia AEs, including hypotension, was low across the treatment groups (ertugliflozin and placebo).

A strength of the analysis is that the very similar design and endpoints of the three primary studies enabled data to be pooled, providing a larger number of patients than in any individual study and enabling an analysis of ertugliflozin efficacy in a variety of patient subgroups. Limitations include the post hoc exploratory nature of the analysis, which meant that no hypothesis testing was planned or performed, the relatively low number of patients on a diuretic, with renal impairment (eGFR < 60 mL/min/1.73 m^2^), and the enrollment of a small number of Black patients. In addition, adjustments for changes to dosing of concomitant antihypertensive therapies were not included in the analysis.

## Conclusion

In conclusion, treatment with ertugliflozin 5 mg and 15 mg over 26 weeks was well tolerated and resulted in reductions in SBP, DBP, pulse pressure, mean arterial pressure, and double product relative to placebo. The SBP effect was consistent across patient subgroups and reductions in BP were achieved without an increase in pulse rate.

## Additional file

**Additional file 1.** List of MedDRA preferred terms for diabetic microvascular complications

## Abbreviations

AE: adverse event
BMI: body mass index
BP: blood pressure
bpm: beats per minute
CI: confidence interval
CV: cardiovascular
DBP: diastolic blood pressure
eGFR: estimated glomerular filtration rate
HbA1c: glycated hemoglobin
LS: least squares
MedDRA: Medical dictionary for regulatory activities
RAAS: renin–angiotensin–aldosterone system
SBP: systolic blood pressure
SD: standard deviation
SGLT2: sodium–glucose cotransporter 2
T2DM: type 2 diabetes mellitus
VERTIS: eValuation of ERTugliflozin effIcacy and Safety
