# Supplementary material for: Effect of ertugliflozin on blood pressure in patients with type 2 diabetes mellitus: a post hoc pooled analysis of randomized controlled trials
Source: Cardiovasc Diabetol. 2019 May 7;18:59. doi: 10.1186/s12933-019-0856-7 (PMC6503446; doi:10.1186/s12933-019-0856-7)
Supplement: Supplementary file 1 — Additional file 1. List of MedDRA preferred terms for diabetic microvascular complications [file 12933_2019_856_MOESM1_ESM.docx]

# Additional materials

## List of MedDRA Preferred Terms for Diabetic Microvascular Complications

Acute painful neuropathy of rapid glycemic control

Acute polyneuropathy

Albumin urine present

Autonomic neuropathy

Chronic kidney disease

Decreased vibratory sense

Demyelinating polyneuropathy

Diabetic end-stage renal disease

Diabetic foot

Diabetic foot infection

Diabetic nephropathy

Diabetic neuropathic ulcer

Diabetic neuropathy

Diabetic retinal edema

Diabetic retinopathy

Diabetic ulcer

Exudative retinopathy

Microalbuminuria

Protein urine

Protein urine present

Proteinuria

Retinal laser coagulation

Retinopathy

Retinopathy proliferative
